# The Impact of Disease Control Measures on the Spread of COVID-19 in the Province of Sindh, Pakistan

**DOI:** 10.1371/journal.pone.0260129

**Published:** 2021-11-18

**Authors:** Bilal Ahmed Usmani, Mustafain Ali, Muhammad Abul Hasan, Amna Rehana Siddiqui, Sameen Siddiqi, Aaron Guanliang Lim, Saad Ahmed Qazi

**Affiliations:** 1 Department of Biomedical Engineering, NED University of Engineering and Technology, Karachi, Pakistan; 2 Centre of Infectious Disease Modeling, NED University of Engineering and Technology, Karachi, Pakistan; 3 Neuro-Computation Lab, National Centre of Artificial Intelligence, NED University of Engineering and Technology, Karachi, Pakistan; 4 Department of Community Health Sciences, Aga Khan University, Karachi, Pakistan; 5 Population Health Sciences, Bristol Medical School, University of Bristol, Bristol, United Kingdom; 6 Department of Electrical Engineering, NED University of Engineering and Technology, Karachi, Pakistan; Nanyang Technological University, SINGAPORE

## Abstract

The province of Sindh reported the first COVID-19 case in Pakistan on 26^th^ February 2020. The Government of Sindh has employed numerous control measures to limit its spread. However, for low-and middle-income countries such as Pakistan, the management protocols for controlling a pandemic are not always as definitive as they would be in other developed nations. Given the dire socio-economic conditions of Sindh, continuation of province-wise lockdowns may inadvertently cause a potential economic breakdown. By using a data driven SEIR modelling framework, this paper describes the evolution of the epidemic projections because of government control measures. The data from reported COVID-19 prevalence and google mobility is used to parameterize the model at different time points. These time points correspond to the government’s call for advice on the prerequisite actions required to curtail the spread of COVID-19 in Sindh. Our model predicted the epidemic peak to occur by 18^th^ June 2020 with approximately 3500 reported cases at that peak, this projection correlated with the actual recorded peak during the first wave of the disease in Sindh. The impact of the governmental control actions and religious ceremonies on the epidemic profile during this first wave of COVID-19 are clearly reflected in the model outcomes through variations in the epidemic peaks. We also report these variations by displaying the trajectory of the epidemics had the control measures been guided differently; the epidemic peak may have occurred as early as the end of May 2020 with approximately 5000 reported cases per day had there been no control measures and as late as August 2020 with only around 2000 cases at the peak had the lockdown continued, nearly flattening the epidemic curve.

## Introduction

In the wake of the COVID-19 pandemic, the nations of the world have exercised strong precautionary measures in order to prevent its spread. Prolonged implementation of these measures is unsustainable from a socioeconomic point of view especially in low- and middle income country (LMIC) settings such as Pakistan [1, 2]. This is due, among other reasons, to a weak healthcare system, limited public health capacity, unpredictable law, order and security issues and a low literacy rate [3]. Commencing from 26^th^ February 2020, COVID-19 gripped Pakistan with more than 295,000 cases to date (28^th^ August 2020). The first two cases of COVID-19 were reported in the province of Sindh located in southern parts of Pakistan. From a population of approximately 50 million, to date (28^th^ August 2020), more than 129,000 cases have been reported from Sindh only. Despite the rapid increase in earlier months and against all predictions, since June 2020, there has been a remarkable reduction in the number of reported COVID-19 cases in Pakistan and in the province of Sindh; the daily number of confirmed cases having reduced from more than around 2000 cases per day during the peak to less than 400 cases per day in August 2020 [4]. Due to the epidemic curve for the first wave now in its receding phase, many theories are being attributed to this decrease; one that merits further consideration is the key role of the Government of Sindh (GoS) in implementing strict control measures (effective from 23^rd^ March 2020). These measures included preventive actions such as closure of schools/offices, restricting mass gatherings, strict lockdown, mask usage, physical distancing, isolation of cases [5] and quarantines [6].

While governments and all partners are wiser and more informed with the enormous experience gained over the last six months globally, earlier on there was no standard or well-defined protocol available for controlling the pandemic in Pakistan, as in many other LMICs. It was then reported that a prolonged lockdown could inadvertently cause economic recession, unemployment, hunger, and aggravate mental health problems and depression within the isolated communities [7]. Thus, it was imperative for the GoS to be well prepared to meet the socio-economic and healthcare needs of the population, should the COVID-19 crisis exacerbate in the region. In this regard, one key aspect of preparedness was to engage in predictive modeling of the course of the epidemic for which the COVID-19 Sindh Monitoring Cell in the GoS collaborated with the Centre for Infectious Disease Modeling (CIDM) at NED University of Engineering & Technology (NEDUET).

Modelling is a powerful tool for studying epidemics and, when combined with robust data, that can generate insightful and reliable predictions. We helped the GoS consolidate its plans for meaningful public health response for tackling COVID-19 in Sindh province. We used data-driven models to provide advice on the most suitable time for implementation of control measures based on estimation the epidemic peak amplitude and timing of the peak in Sindh [8]. Not unlike other studies, we also assessed the impact of imposition of lockdowns especially in the context of LMICs [9]. This study displays how the results of predictive modeling evolved with the data as it was collected over time and how the incorporation of different disease control measures and public response into the modeling paradigm affected key epidemiological parameters such as the *final size* of the epidemic and the *peak time* of epidemic. Simultaneously, social events such as religious congregations and observances typical to the Islamic culture; such as Ramadhan and Eid festivities, were also notable in the spread of COVID-19. It is shown in this paper how these interventive measures were likely to be effective in mitigating the spread of COVID-19 in Sindh by predicting its short-term dynamics [10].

This paper first describes the data used in this study and then provides a walkthrough of the model structure, assumptions, the modeling timelines along with their associated events followed by the model parameters and analyses in the methods sections; the section following the methods reports and discuss the main findings of this study.

## Materials and methods

### Data description

The COVID-19 Sindh Monitoring Cell provided the CIDM, NEDUET special access to a dashboard maintained daily for COVID-19 patients in Sindh. The data for the daily reported cases from the beginning of the epidemic till the timeline at which the modelling is performed was taken from the GoS website/dashboard. In addition to that, the daily number of recoveries and deaths were also taken from the same dashboard.

### Model structure

For modelling the effect of GoS measures on COVID-19 spread, a minimalist SEIR modelling structure was used in this study. The simple SEIR model used explicit compartments that helped model the latent or exposed individuals as COVID-19 is believed to have an appreciable incubation time of around 2 to 8 days prior to the onset of active infection [11–14]. The model considered the population into four broad, non-overlapping compartments according to their health status: healthy individuals that may contract the infection (Susceptible—S); individuals that are infected but not infectious to others (Exposed—E); Infectious individuals (Infected—I); and individuals who have recovered and are now immune or have been Removed through death (Removed—R). The model comprises a set of ordinary differential equations (Eq 1–Eq 4) corresponding to the rate of change of susceptible, infected exposed and removed individuals.


$$\dot{S}=-\beta SI.$$ (1)

$$\dot{E}=\beta SI-\sigma E.$$ (2)

$$\dot{I}=\sigma E-\delta I.$$ (3)

$$\dot{R}=\delta I.$$ (4)

### Model parameters

The model parameters comprise the transmission rate *β* at which an individual acquires new infection and becomes exposed to the disease. Until the onset of infectiousness, the individual is in a latent or incubation phase during which the viral load increases. The next transition occurs at an exposure rate *σ* at which an exposed individuals develops clinical symptoms of COVID-19 and becomes infectious. An individual ceases to be infectious after making a complete recovery from the disease at a recovery rate of *δ*.

### Model assumptions

Standard assumptions have been made for the simple SEIR epidemiological models without demography [15] for further details of the SEIR model We make the following assumptions:

- With only Sindh considered as a region of interest in this study, it was assumed that there would be a negligible change in the overall demography within the span of a year.
- With a very low burden of disease induced deaths in Sindh (as seen from the Sindh monitoring cell dashboard), it was not incorporated into the modeling timeline.
- Out of the total population of Sindh, a maximum of 0.6% would get infected during the first wave of the epidemic.
- The closure assumption of the host population (H) leads to H = S + E+ I +R.
- The exposed and infectious period of an individual is exponentially distributed as $\frac{1}{\sigma }\&\frac{1}{\delta }$ respectively.
- The model assumes homogenous population mixing however, with lockdowns and limited human movement due to lockdown, the disease transmission event has been correlated with the mobility of people during the epidemic timeline. (Discussed below)
- Studies also suggested that massive under reporting [16] and a significant proportion of missed cases [17] present in the population were the major contributors to the COVID-19 burden. According to the Centre for Disease control, (CDC, USA), for every reported case there were ten times more true cases [18]. We have taken this assumption to estimate the actual cases in Sindh on the basis of reported cases. Evidence from other settings appear to suggest that the prevalence of asymptomatic infections needs to be reported at a higher magnitude, around 45% to 50% percent of the reported cases [19, 20]. In addition to that, our study assumes that the under reporting, mainly due to lack of testing facilities and partly due to fear of disclosure among people [21, 22] yields to at least 40–50% of the missing cases. In absence of any other study in population of Sindh for gauging proportion of asymptomatic infections during the first wave, this provides a basis for taking the estimate provided in [18].

### Modeling timelines

A series of runs of the SEIR model were carried out for different timelines which correspond to the following events and their effects on the spread of COVID-19 in Sindh:

1. **Timeline 1**: **26**^**th**^ **Feb to 26**^**th**^ **March 2020** from the beginning of the epidemic using data from Wuhan,
  ➢ Province wide Lockdown, closure of schools, restaurants, etc., banning of public gatherings, congregational prayers, religious processions etc. (effective: 23^rd^ March 2020 onwards)
2. **Timeline 2**: **26**^**th**^ **Feb to 5**^**th**^ **April 2020** during lockdown implementation by the GoS,
  ➢ Increased Testing Services/Reporting (effective: 10^th^ April onwards)
3. **Timeline 3: 26**^**th**^ **Feb to 10**^**th**^ **May** after relaxation of the imposed lockdown,
  ➢ Relaxation of Lockdown/ Partial Lockdown from 9^th^ May 2020 onwards
4. **Timeline 4: 26**^**th**^ **Feb to 31**^**st**^ **May** after the commemoration of Eid-ul-Fitr* festivities,
  ➢ Religious event after the Islamic month of Ramadhan during which celebrations take place; Shops and Markets were opened for preparations with mass gatherings on Eid (effective: last two weeks of May 2020)
5. **Timeline 5: 26**^**th**^ **Feb to 11**^**th**^ **August 2020** After the commemoration of Eid-ul-Azha* festivities (* Religious Events celebrated in the Islamic World during which mass gatherings and congregations take place).
  ➢ Religious event after Hajj pilgrimage caused even more mass gatherings, Shops, markets, and workplace open (effective: Last week of July to First Week of August 2020) whereas restaurants opened from 10th August 2020.

These specific dates and time periods correspond to the GoS’s call for advice from CIDM on the prerequisite actions required to curtail the spread of COVID-19 in Sindh. Looking back on them now provides insight on the impact of the control actions taken by the GoS and the subsequent population response.

### Choice of parameter values

**Fig 1:**
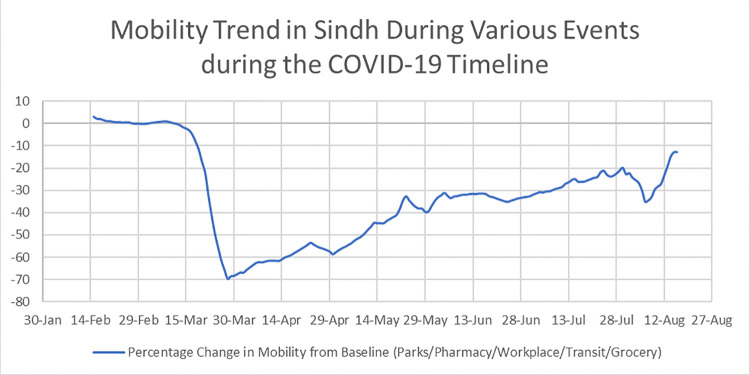
The mobility patterns recorded in Sindh during the period of January to August 2020. Dataset have been taken from publicly online database (www.google.com/covid19/mobility/). This figure depicts the percentage change in mobility during the period of 30^th^ Jan to 16^th^ August 2020. Mobility patterns have been recorded for people leaving their residence for visiting parks, pharmacies, workplace, bus or train stations and for grocery shopping.

The value for *β* was estimated for each of the modelling timelines stated above. The GoS asked the CIDM for guidance before taking public health decisions whereas the transitioning rates were estimated from existing literature, as shown in Table 1. The estimation of the transmission parameter was done by using the prevalence data and verified by using the global mobility report downloaded from the publicly available google database (www.google.com/covid19/mobility/). The graph in Fig 1 has been plotted using MS Excel (2019) and it describes the google mobility trend categorized as the percentage of people leaving their homes for visiting the following places: pharmacies, places for buying groceries, parks/recreational places, transit points (Bus/train stations, etc.) and workplaces.

**Table 1:** The table shows the parameter values used in the SEIR model, parameter values of transmission rate have been calculated from the accuracy of fitted data curves whereas the incubation rate and the recovery rate have been averaged out to 7 and 8 days respectively based on the values obtained from various literature.

Parameter	Symbol	Timeline 1 (26^th^ Feb to 26^th^ Mar)	Timeline 2 (26^th^ Feb to 5^th^ April)	Timeline 3 (26^th^ Feb to 10^th^ May)	Timeline 4 (26^th^ Feb to 31^st^ May)	Timeline 5 (26^th^ Feb to 11^th^ August)	Source
Transmission Rate (*days*^−1^)	*β*	0.4	0.235	0.265	0.28	0.3	Data fitted Values
Average Incubation Rate (*days*^−1^)	*σ*	$\frac{1}{7}$	$\frac{1}{7}$	$\frac{1}{7}$	$\frac{1}{7}$	$\frac{1}{7}$	[11–14]
Average Recovery Rate (*days*^−1^)	*δ*	$\frac{1}{8}$	$\frac{1}{8}$	$\frac{1}{8}$	$\frac{1}{8}$	$\frac{1}{8}$	[23–26]
Decrease in Mobility from Baseline	---	Baseline (0%)	−63.5% (36% mobility)	−48% (52% Mobility)	−37% (63% Mobility)	−27% (73% Mobility)	Google Mobility Data
Percentage Adjustment for *β* based on Data fitting	---	---	59% Mobility	66% Mobility	70% Mobility	75% Mobility	Data Fitting

Using MS Excel, a moving average (a series of averages of different subsets of the full data set) of all five categories (of the mobility trends mentioned above) was taken to represent the overall effect of the control measures like lockdown and other events (mentioned below) on the movement trends in Sindh throughout the major course of the epidemic. The negative peaks in the Fig 1 have been correlated with the various events which occurred during the epidemic timeline.

The same model structure has been used for all five timelines modelled however, the parameter values changed with the progression of the epidemic. The effects of the model parameters on the model outcomes have been discussed through changes in the peaks and amplitude of the infectious cases. The initial value of transmission rate for the very first timeline was taken to be 0.4 *per day* which was analogous to the transmission rate in Hubei at the early time of the epidemic. For all subsequent modelling timelines made, the transmission rate was subjected to finding the best-fit curve from the data of daily reported cases data in Sindh and linked with the change in mobility found from the Google mobility data. The average incubation periods were taken as 1/7 days^-1^ and 1/8 days^-1^ respectively. As seen in literature, the incubation period and infectious period have a large range. Univariate sensitivity analysis (see S1 File) was carried out for the incubation and the recovery rate against the different adjusted transmission rates. The model parameter value evolution according to the timelines have been mentioned in the Table 1 below.

Table 1 describes the parameter values used in the model and shows which values of the transmission parameter used during each timeline. Then it mentions the percentage mobility extracted from the google mobility dataset. For each timeline modeled during the COVID-19 outbreak, there was a marked reduction in the mobility of the population in Sindh; the decrease is shown as a negative percentage when measured from the baseline. Contrastingly, the positive percentage increase in mobility is also mentioned with the passage of time. The adjustment of *β* has been made along the lines of this positive percentage.

### Model analyses

Using MATLAB (version 2020a), the transmission potential of COVID-19 was estimated using the basic reproductive number R_0_. It is a quantitative representation of the average number of secondary cases produced by an infected individual, during the lifetime of infection, in a naïve/totally susceptible population. Similar to other studies, the general form of R_0_ was calculated by the next-generation matrix approach [27, 28]. The model simulated data from the beginning of the epidemic up to the current data fed into the model; the R_0_ was evaluated and reported each time the solver was run for the different epidemic timelines.

### Results

**Fig 2:**
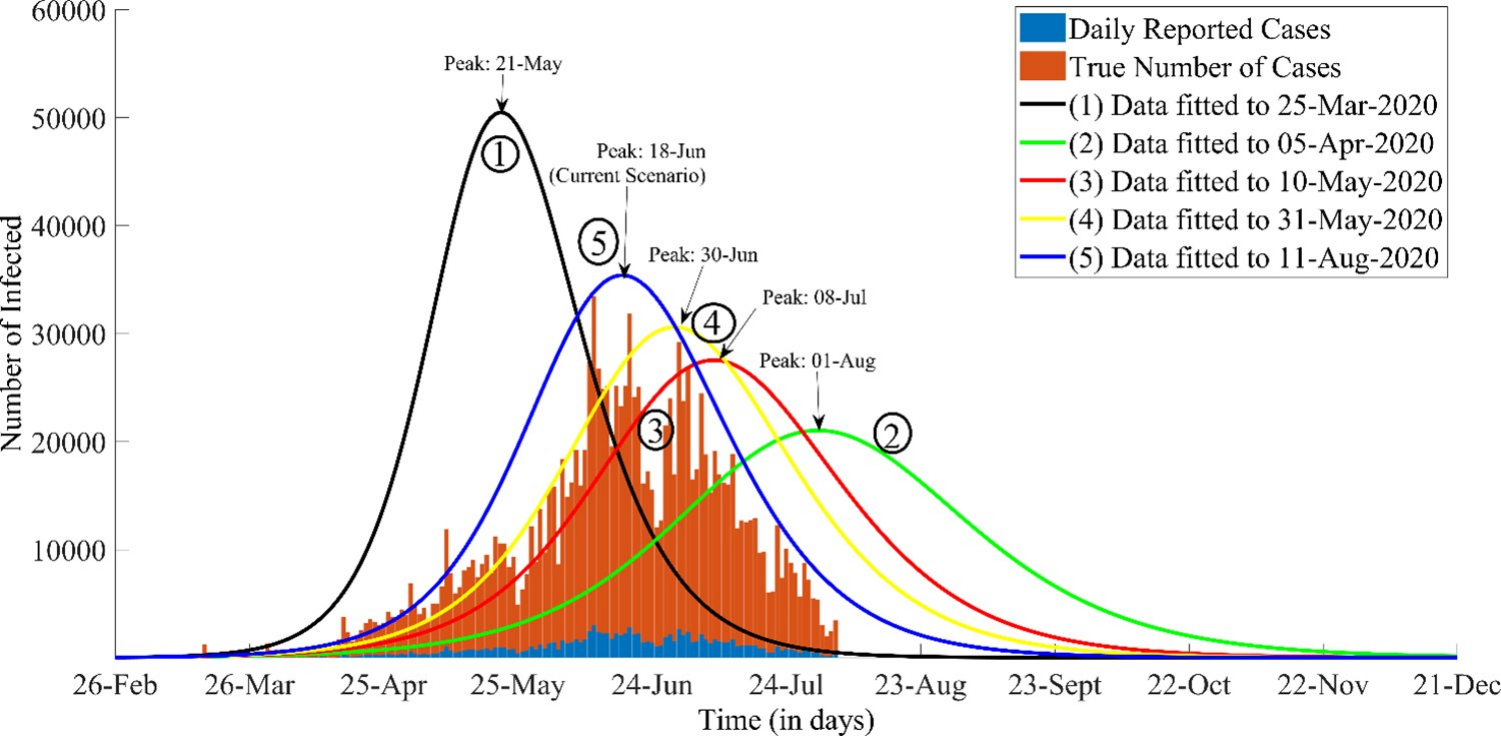
The Evolution of the COVID-19 modelling paradigm for different epidemic timelines in Sindh. The labelled graphs depict the number of infected individuals throughout the course of the epidemic. The curves correspond to the following important events: 1. The initial model outcome was based on the parameter values obtained from data for Wuhan, China and shows an unprecedented growth of the epidemic. 2. Initial Lockdown and community sensitization was more effective. The green plot depicts the most feasible outcome, had the preventive trend continued. 3. Testing Capacity increased from beginning of April onwards which is why more cases were being reported, shifting the data curves fitted till day 60 (red) further to the left of green curve. 4. A further surge in transmission was observed after 9th May was due to ease in lockdown, which pushed the peak of the yellow plot around 17% backwards. 5. It is evident from the blue plot (latest results) that due to the recent Eid celebrations and re-opening of non-essential businesses, the curve has moved further backward consequently hastening the peak of the epidemic Note: The blue stacked bars represent the daily reported cases and the brown stacked bars reflects the model assumption of having 10 times more (actual) cases than reported.

The consolidated results of the predictive SEIR model for the five different timelines are displayed in Fig 2 below, with a more detailed description in Table 2. The model results project the size, duration and peak of epidemic. The fully plotted data is only relevant to modeling timeline 5 as this was the latest model to date (11^th^ August 2020).

**Table 2:** A summary of the results obtained based on the governmental measures taken and its impact on the model through variation in the transmission rate *β*.

Local Events and Government Measures	Modeling Timeline	*β*	R_0_	Peak	Number of Cases per day at Peak	% Amplitude Shift in Peak Position	Time Shift in Peak Position
• None	26^th^ February to 26^th^ March (Day 30)	0.4	3.2	21^st^ May 2020 (Day 86)	5047	---	---
• Province wide Lockdown	26^th^ February to 5^th^ April (Day 40)	0.235	1.88	1^st^ Aug 2020 (Day 158)	2104	**Difference from Timeline 1**	
• Closure of Schools, restaurants, etc.						−139%	+72 Days
• Banning of Public gatherings, congregational prayers, religious processions etc.							
• Increased Testing Services/Reporting	26^th^ February to 10^th^ May (Day 74)	0.265	2.12	8^th^ July 2020 (Day 136)	2755	**Timeline 3 vs Timeline 1**	
• Lockdown relaxed (due to economic stress and public unrest) on weekdays till 5:00 pm and complete lockdown on weekends						−83.19%	+50 Days
						**Timeline 3 vs Timeline 2**	
						+23.62%	−22 Days
• Religious event celebrations (Eid-ul-Fitr festivities), Shops and Markets Opened for preparations	26^th^ February to 31^st^ May (Day 95)	0.28	2.24	30^th^ June 2020 (Day 126)	3060	**Timeline 4 vs Timeline 1**	
						−64.93%	+40 Days
						**Timeline 4 vs Timeline 2**	
						+31.24%	−32 Days
• Mass gatherings on Eid						**Timeline 4 vs Timeline 3**	
						+10%	−10 Days
• Public unrest causing blatant disregard for the lockdown	26^th^ February to 11^th^ August (Day 161)	0.3	2.4	18^th^ June 2020 (Day 116)	3449	**Timeline 5 vs Timeline 1**	
						−46.33%	+30 Days
• Eid-ul Azha festivities (Religious event after Hajj pilgrimage) caused even more mass gatherings						**Timeline 5 vs Timeline 2**	
						+39%	−42 Days
						**Timeline 5 vs Timeline 3**	
						+20.12%	−20 Days
						**Timeline 5 vs Timeline 4**	
• Shops, markets and workplace open						+11.27%	−10 Days
• Restaurants to open from 10^th^ August 2020							

Fig 2 has shown how the epidemic peak would have looked like assuming each trend was maintained without changes made past each fitted date. With the passage of time, the model fit eventually gets better and the fifth modeling timeline shows the best fit to the data. Each plot in the figure has been plotted separately as well to see the “goodness” of the fit for each modeling timeline with the reported data at the time of which it was made. (See S1 File)

Table 2 quantitatively describes the effect of community sensitization to COVID-19 and the public unrest due to the control measures implemented by government through shift in the peaks of the epidemics (Fig 2). The calculated R_0_ values are also listed along with continual change in the reproduction number as the modeling timelines change. As for each modeling timeline the value of *β* has been fitted to the mobility data; this evolution of the transmission parameter was reflected in the epidemic size and the duration of epidemic. For a larger value of transmission, the epidemic was short lived and peaked quickly with a relatively high epidemic size. With changes in the transmission, the peak shifted. This shift in peak has been described in the table as the percentage amplitude shift in the peak. Since it was assumed in the study that the true cases were ten times more than the reported daily cases, the column “number of cases per day at the peak” were reduced ten times to show correlation with the daily reported cases in Sindh. The basic reproductive number was calculated for each timeline. The time shift in peak position describes the duration of COVID-19 given the control measures being implemented. During the lockdown phase, the time to reach the epidemic peak was projected in the first week of august (1^st^ August 2020). Moreover, the modeling timelines have been compared with one another and described quantitatively in terms of the percentage increase/decrease and the number of days reduced or added in reaching the peak.

## Discussion

Using the results from the model, the peak amplitude and timing of the peak were being communicated to the GoS to plan for the COVID-19 public health response in Sindh. The evident shift in the epidemic peak corresponded to the social and religious events that were taking place in the province, sometimes compromising the effectiveness of the measure taken to curb the spread. Fig 2 depicted the impact of these interventions both quantitatively and somewhat, qualitatively as well. The percentage depreciation of the peak and the time shift of the peak in Table 2 suggests that limiting human contact could have an impact on controlling disease spread as each time a potential super-spreader event such as a mass-gathering or religious procession takes place; such events are reflected onto the transmission rate and there is a shift in the peak of the epidemic. This impact can be further corroborated by means of evaluation of the effective reproductive number (R_e_) which can account for the depletion of the susceptible pool and the effect of community sensitization that promotes behavioral changes such as inhibiting oneself from human contact. However, since only a fraction of the population has been assumed to be infected. It seems that the evaluation of R_e_ would be done in future models by employing stochastic modeling techniques and estimating R_0_ driven through human contact patterns. The concept of a spatially evaluated reproductive number may prove to be a better approximation for the disease.

The google mobility data sheds light on the conditions of Sindh during the imposed lockdown. During the lockdown, the essential businesses remained open for restricted working hours. Some of the businesses were opened from 9 am to 5 am, providing limited access to the people. Intuitively high-income population groups could stock household supplies in response to the imposed lockdown. Meanwhile, reports from all over the country came flooding in when the lower-income groups were forced to come out of their homes especially the daily wage earners and others in times of supplies need [29–32]. We assumed that the “percentage of people leaving the house for grocery” would be the main indicator for human mobility whereas the secondary indicators were “leaving for work place, leaving for transit stations and retail and recreation”. The rationale behind this was that people would undoubtedly have to leave their homes to replenish their household supplies. We correlated this movement with the number of people leaving their homes. A positive association can be seen between the dates, the percentage of people leaving their homes, the modeling events, and the transmission rate. The GoS have implemented numerous public health interventions mostly on community movement and interactions to curtail COVID-19 transmission in Sindh. Since, the governmental control actions were focused on restricting human movement; it was theorized that the movement of a significant amount of people would contribute to increasing disease transmission given the assumption of homogeneous population mixing. With the implementation of lockdown, the mobility trend gradually reduced from the base line but with the passage of time this trend evolved as is evident from Fig 2.

Pakistan lacked data till the 25^th^ day of epidemic as no time series had been built by then hence data from Wuhan, China was chosen as a reference for the initial modeling timeline only and for the initial values of parameters in the model. Additionally, given the fast spread of COVID-19 in Sindh, several cases for the first model (not shown here) were made in which the transmission dynamics was studied under 10%, 25% and 50% infectious spread in a population. However, with further accumulation of time series datasets (publicly available online) for different countries, a trend was observed that only mere fractions were getting infected. When the first wave of COVID-19 had passed in China, it showed that only 0.0058% of Chinas population had been infected throughout the course of the first wave. For Italy it was 0.44%, Thus, from the second modelling timeline onwards, the model design assumed that only 0.6% of the total population of Sindh (*for N* = ~50,000,000) would be infected during the first wave of COVID-19 spread in Sindh. This assumption acts as an upper bound of infection spread in the population of Sindh, pertaining to the proportion of international cases across the globe.

In this study we have used a minimalist modeling [33] approach to manage the rates of transmission, recovery and exposure rates to inform public health policy responses due to limited or no data being available on asymptomatic carriers, age stratified cases and other such heterogeneities. Thus, the modeling paradigm employed for this study steadily evolved. Given the complexity of the disease trajectory, it is important to include severity of disease as well as stratification of the model by age (which also requires knowledge of contact patterns by age) but with difficulty in obtaining such disaggregated datasets and huge uncertainties associated, they were not incorporated into this model. Nevertheless, our model produces projections that agree well with the data, so provides a good representation of the COVID-19 epidemic in Sindh despite its relative simplicity. As more data become available, this will allow us to parameterize more complex models that can capture more detailed aspects of the COVID-19 epidemic in Sindh. Early in the epidemic, several key epidemiological features were still unknown and there was a certain degree of uncertainty. To date, there is still a lack in the complete understanding of the contextual factors that can influence the transmission and affect the disease severity, especially in a LMIC setting.

Since this is a simplistic model, it only has a single infectious compartment, however, infectious individuals may be either pre-symptomatic (infected individuals who are bound to develop symptoms) [34], asymptomatic (infected individuals whom will not develop symptoms of infection) [35, 36] or symptomatic. Moreover, our model uses a rough estimate for the asymptomatic cases which may not be the case. It was reported for China that four-fifths of the COVID-19 burden was due to the true asymptomatic infections [37] whereas in other literature it was estimated to be less than half of the confirmed cases [38]. In the beginning, the asymptomatic burden was not incorporated into the model but from the second modeling timeline onwards to all subsequent timelines, data fitting included fitting reported cases data to ten times more cases (by means of stacked histograms shown in Fig 2) which were estimated to be the true number of cases. It seems like a reasonable assumption given the fact that Sindh has an over-burdened health infrastructure with limited testing facilities and the nation has been unable to meet the WHO goal of carrying out 50,000 tests per day.

Homogenous mixing of population has been assumed for this study and contact patterns have not been evaluated which essentially can paint a very realistic image of the existing chains of infections Additionally, the evaluation of the true value of R_0_ through contact tracing may be performed in the future which would incorporate network dynamics as well. We can also extend the model to include stochastic simulations and observe the long-term dynamics of COVID-19 [39, 40].

## Conclusions

Since the pandemic began seven months ago, there has been a surge of information provided to help shape our models and to provide better predictions. The teams that were collecting data on ground and our modeling results complemented with their acquired data. The GoS has used the model outcomes to guide public health policy in Sindh. The outcomes of these models helped guide the GoS to take timely and necessary actions based on the predicted peak and the burden of cases on the peak. Even though, the model outcomes suggest that limiting human contact had an impact on the movement of peak ahead in time and the consequent flattening of the epidemic curves, it has also been inferred that even during a pandemic, the economic stress and the subsequent public unrest can hinder the efficacy of the governmental actions taken to mitigate the COVID-19 spread in Sindh. For poverty-stricken communities, hunger takes precedence over following standard control procedures. There is an urgent need to address and resolve the issues faced by such communities. According to local news reports, the government of Sindh has taken an initiative by providing rations and monthly stipend to the daily wage earners. The government of Sindh can also benefit by following the steps taken by other countries to maintain national stability through governmental actions; the Chinese government supplying groceries at the doorsteps of isolated citizens, or the Italian government providing monetary assistance to boost economy and keep the businesses up and running are just a few examples.

## Supporting information

S1 File (DOCX)
