# Supplementary material for: The Impact of Disease Control Measures on the Spread of COVID-19 in the Province of Sindh, Pakistan
Source: PLoS One. 2021 Nov 18;16(11):e0260129. doi: 10.1371/journal.pone.0260129 (PMC8601461; doi:10.1371/journal.pone.0260129)
Supplement: S1 File — (DOCX) [file pone.0260129.s001.docx]

# Supplementary Information

## Uncertainty Analysis

A crucial aspect of modeling infectious diseases is the estimation of epidemiological parameters. Using outbreak data for COVID-19, the parameters are usually estimated via techniques that can fit model to data. Uncertainty analysis is performed on these fitted data sets to account for any errors that may arise from noisy or faulty data.

With the presence of a latent class in the SEIR model for COVID-19, the average latency period and average infectious period are assumed to be exponentially distributed with a duration of $\frac{1}{\sigma}$ and $\frac{1}{\delta}$, respectively. It is evident from the figure below that the higher values of average latency will affect the amplitude of the peak as well as delay the start of COVID-19. This is because it will take a longer time for the actual infection to begin and thus, the reproductive number would be greatly reduced as well. We assumed that the parameters are sampled from an exponential distribution and the choice of baseline values were 1/7 for exposure and 1/8 for recovery respectively [1, 2]. A numerical solver (ode15s) was employed to evaluate the plots and the MATLAB interface was utilized to run the program and solve the system of differential equations. The results of which are displayed in S1 Figs 1 and 2.


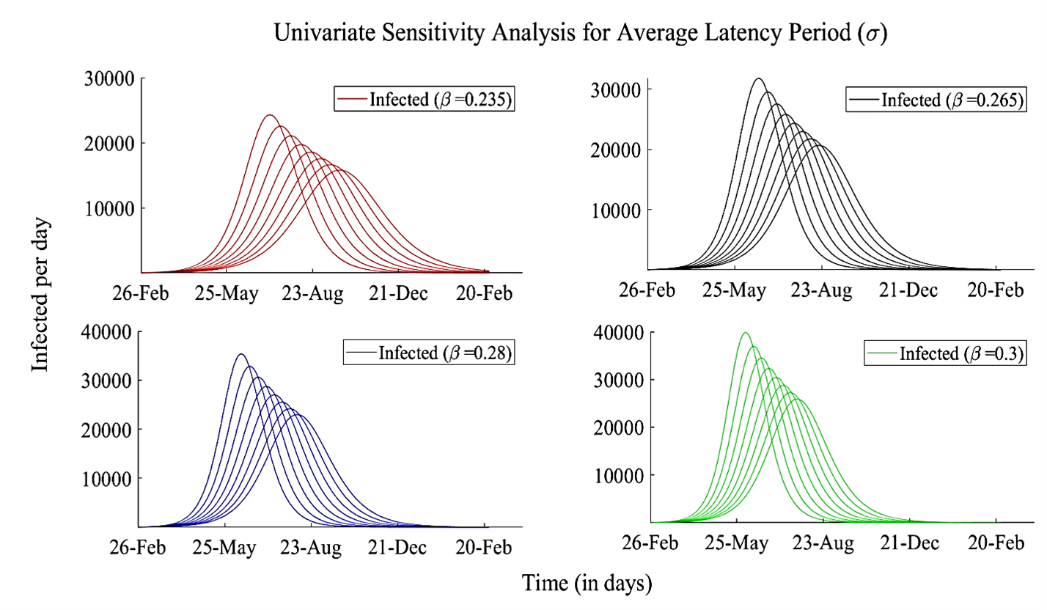


**S1 Fig 1. The long-term projection of COVID-19 cases occurring per day in Sindh for four different timelines with varying Latency period**

Each timeline had a separate transmission parameter (β) which was obtained via google mobility data and adjusted by fitting data to the model. These plots represent the variation in the average incubation rate (1/σ) for each timeline using baseline values for average recovery and transmission rate. The minimum value of incubation in literature has been found to be 5 days where as a maximum of 14 days has also been reported. The left most plot depicts the infectious cases if the average incubation period is 5 days, otherwise the subsequent plot shows infectious cases for $\sigma=6$, then $\sigma=7$and similarly consecutive plots have been made up to $\sigma=12.$ The changes in σ considerably delay the epidemic peak and subsequently increase the prevalence of COVID-19 in the population.


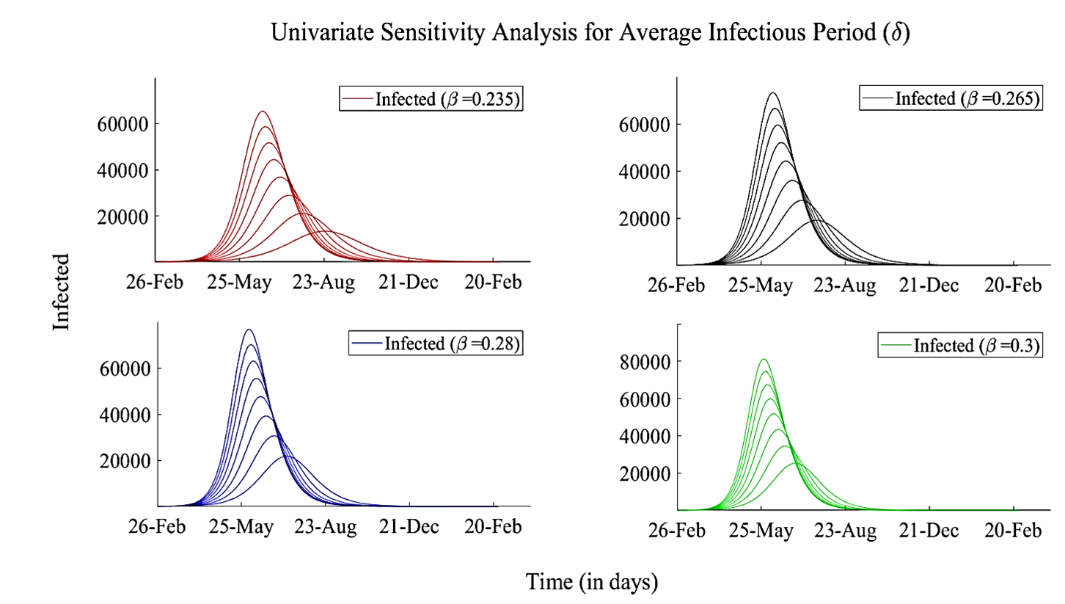


**S1 Fig 2. The long-term projection of COVID-19 cases occurring per day in Sindh for four different timelines with varying average infectious period**

Each timeline had a separate transmission parameter (β) which was obtained via google mobility data and adjusted by fitting data to the model. These plots represent the variation in the average recovery rate (1/δ) for each timeline using baseline values for average incubation and transmission rate. The minimum value of average duration of infectiousness in literature has been found to be 7 days where as a maximum of 12 days has also been reported. Similar to the previous plots, the left most plot depicts the infectious cases if the average infectious period is 7 days, then the subsequent plot shows infectious cases for $\delta=6$, then $\delta=7$and similarly consecutive plots have been made up to $\delta=14.$. The changes in $\delta$ considerably reduce the final size of the epidemic and the greater the recovery rate, the quicker disease dies off mostly before the end of the year.

Additionally, the reproductive ratio for this model was found to be $R_{0}=\frac{\beta}{\gamma}$. The parameter β is responsible for the progression to an infectious state. The model considered in this study is a standard epidemiological model used in many studies and is locally and globally asymptotically stable for values of parameters in the feasible region: $\left\{ \left( S,E,I,R \right) \epsilon\mathbb{R}_{+}^{3} \right|0\leq S+E+I+R\leq H\}$ [3]

**Modelling attempts for COVID-19**

This section provides a walkthrough for a series of activities carried out in mathematical predictive modeling during the beginning of the pandemic. This work was caried out as a joint-team collaboration between The Centre for Infectious Disease Modeling at the NED University of Engineering & Technology and the Department of Community Health Sciences at the Aga Khan University.

**Models for Hubei, China**

With the initial wave of the pandemic beginning to spread around the world in early 2020, our team began with preparing an epidemic model for Hubei, China. Little was known that these efforts would soon be utilized for studying the same epidemic in Pakistan. The model discussed in this study was developed for Hubei on 6th February 2020. However, it’s parameterization was done by using the time-series of infected and recovered patients from China. This model greatly over-estimated the incidence of COVID-19.


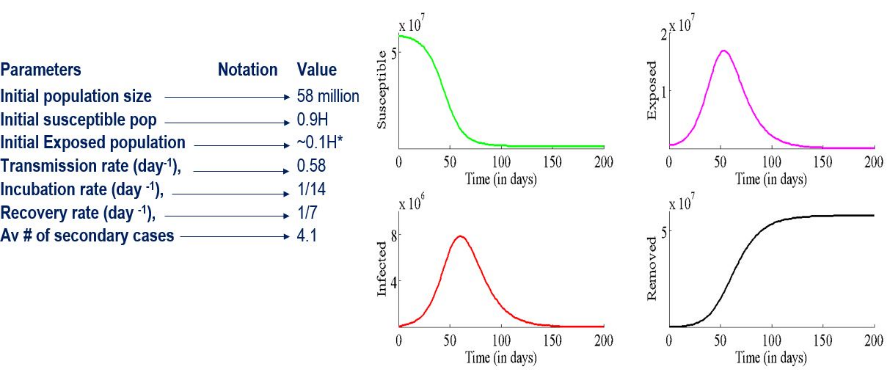


**S1 Fig 3. The estimated spread of COVID-19 in Hubei during the beginning of the pandemic crisis.**

Due to the rising number of cases being reported, 10% of the transmission parameter fit to the data was estimated at $\beta=0.58,$the population of the province that was considered exposed to the disease was 10% with an R_0_ of 4.1.


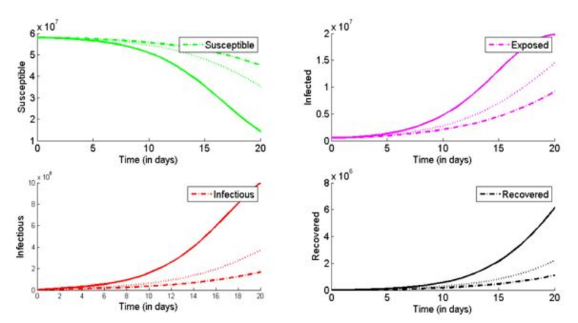


**S1 Fig 4. Trends in the epidemic profile of Hubei, China.**

Fig 4 represents the variation in the incubation rate (σ) of COVID-19. The solid line corresponds to average incubation period = 6 days, the dotted line represents the situation when average incubation period = 10 days & the dashed dot line shows the values of S, E, I and R when average incubation period = 14 days. In all of these plots, the value of R_0_ ranges from 3.8 to 8.

**COVID-19 in Pakistan**

The first case of COVID-19 was reported in Pakistan on 26^th^ February 2020. It originated in Sindh, Pakistan. The modeling and simulation of the epidemic spread in Pakistan and Sindh commenced from 21st March (25th day of epidemic in Pakistan).


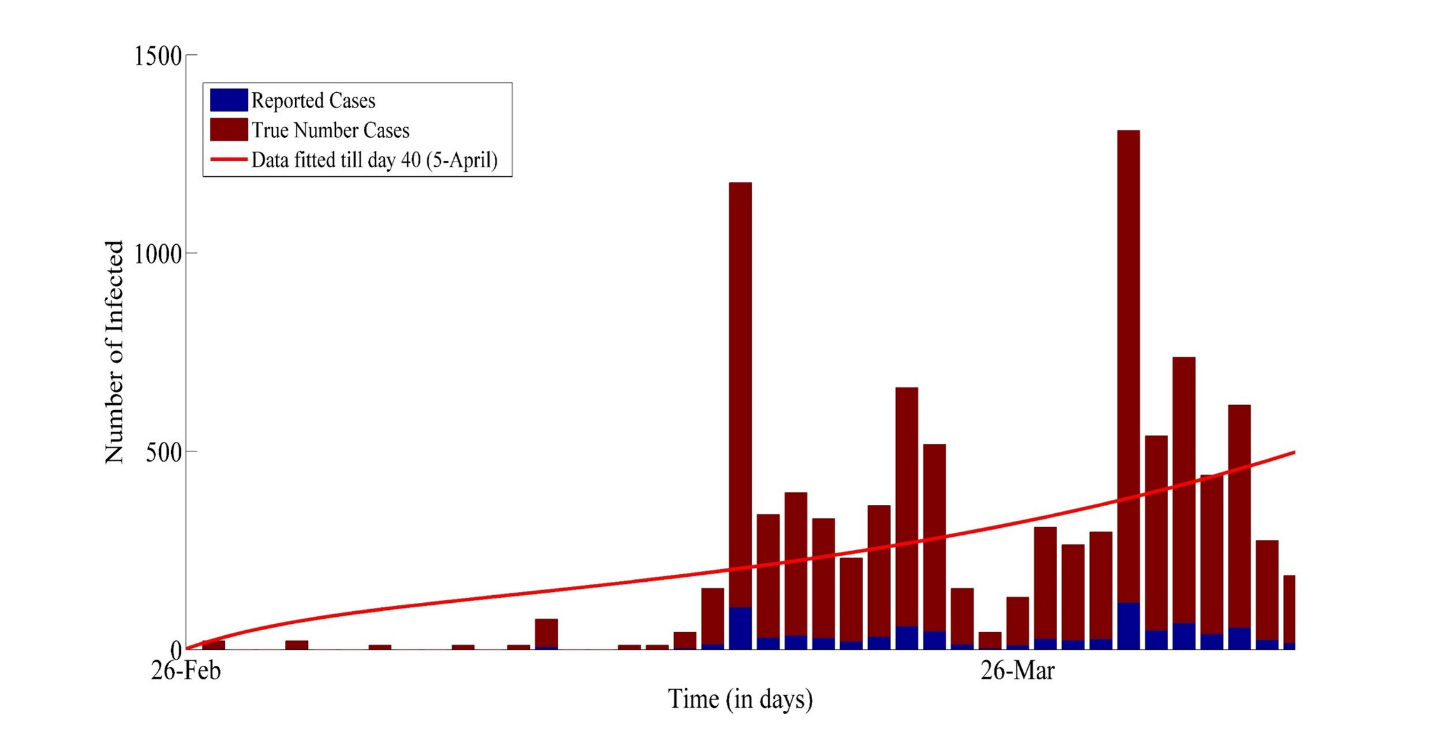


(a)


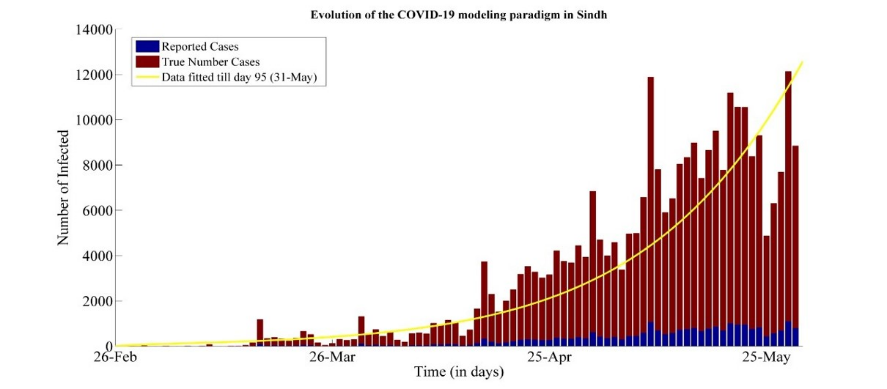


(b)

**S1 Fig 5. The evolution of the epidemic profile through fitting of transmission parameter** $\boldsymbol{\beta}$

Fig 5(a) shows how during the initial wave of the pandemic, small-timescale predictions were being made based on the frequency of reported cases. Using the limited data at the time, we fitted the transmission parameter. Massive under-reporting, limited testing services and the burden of asymptomatic cases was a major factor in making the assumption that the actual burden was ten-folds more than what was being reported. Hence the data-fitting was based on the true burden whereas Fig 5(b) shows the epidemic progress and how the projections improved with accumulation of more data on COVID-19.

The modelling attempts were also made on 60th and 75th day of epidemic in Sindh (shown at the end). After 5th April, modelling was only done for Sindh. This was done to improve parameter estimations, in order to better project the number of cases at different time points including the peak. The reduction in number of cases during lockdown, etc. were translated into the model in terms of fitting of the transmission parameter which gave birth to the concept behind our current study. It stemmed from observation of these evolving plots as to how the control actions changed the epidemic profile upon each modeling attempt. Thus, we ensued a comparative analysis of these plots on how the policy changes affected the transmission dynamics of COVID-19 in Sindh, Pakistan.

**References**

1. Altaf A, Deeba F. Current Scenario of Covid-19 with Epidemiological and Phylogenetic Analysis of Pakistani Coronavirus : A Review Current Scenario of Covid-19 with Epidemiological and Phylogenetic Analysis of Pakistani Coronavirus : A Review. 2020;7(3).

2. Zia K, Farooq U. COVID-19 Outbreak in Pakistan: Model-Driven Impact Analysis and Guidelines. 2020;1–14.

3. Blackwood JC, Childs LM. An introduction to compartmental modeling for the budding infectious disease modeler. Lett Biomath. 2018 Dec;5(1):195–221.
